# Particle Swarm Optimization-Based Support Vector Regression for Tourist Arrivals Forecasting

**DOI:** 10.1155/2018/6076475

**Published:** 2018-09-19

**Authors:** Hsiou-Hsiang Liu, Lung-Cheng Chang, Chien-Wei Li, Cheng-Hong Yang

**Affiliations:** ^1^Department of Tourism Management, National Kaohsiung University of Science and Technology, No. 415, Jiangong Rd., Sanmin District, Kaohsiung 807, Taiwan; ^2^Department of Electronics Engineering, National Kaohsiung University of Science and Technology, No. 415, Jiangong Rd., Sanmin District, Kaohsiung 807, Taiwan; ^3^Graduate Institute of Clinical Medicine, Kaohsiung Medical University, Kaohsiung 80708, Taiwan

## Abstract

The tourism industry has become one of the most important economic sectors for governments worldwide. Accurately forecasting tourism demand is crucial because it provides useful information to related industries and governments, enabling stakeholders to adjust plans and policies. To develop a forecasting tool for the tourism industry, this study proposes a method that combines feature selection (FS) and support vector regression (SVR) with particle swarm optimization (PSO), named FS–PSOSVR. To ensure high forecast accuracy, FS and a PSO algorithm are employed to, respectively, select reliable input variables and to identify the optimal initial parameters of SVR. The proposed method was tested using a data set of monthly tourist arrivals to Taiwan from January 2006 to December 2016. The results reveal that the errors obtained using FS–PSOSVR are comparatively smaller than those obtained using other methods, indicating that FS–PSOSVR is an effective method for forecasting tourism demand.

## 1. Introduction

The tourism industry is one of the fastest promising economic sectors worldwide. Statistics from the World Tourism Organization [1] indicate that the number of inbound tourists increased by approximately 250 million between 2000 and 2010; moreover, this number is predicted to increase to 1.8 billion by 2030. Statistics from the World Travel and Tourism Council (WTTC) [2] show that the tourism industry accounts for 3.4% of world gross domestic product (GDP), and the tourism industry accounts for 2.8% of total employment globally. These statistics indicate how influential the tourism industry is in the global economic environment. The WTTC results related to Taiwan reveal that tourism generated 4.3% of its total GDP in 2017, and, furthermore, workers in this industry accounted for 5.2% of total employment [3]. These statistics indicate that the tourism industry has also become a critical component of the Taiwanese economy. Accurate forecasting of tourist volume plays a major role in tourism planning because it enables destinations to predict requirements for infrastructural development in order to meet demand. In addition, accurately forecasting tourist arrivals and studying tourist arrival patterns are essential measures for tourism-related industries seeking to formulate efficient and effective strategies for maintaining and boosting the tourism sector.

Brida, Cortes-Jimenez, and Pulina [4] mentioned that, in several cases, a long-run bidirectional Granger causality exists between tourism and GDP; therefore, when testing tourism-led growth hypotheses, researchers should aggregate origin countries that exhibit similar features to avoid biased results. Misleading results may emerge in both the short and long term, because various source market segments may possess diverse characteristics. Thus, it may not be valid to state that expansion of the tourism sector contributes to long-term growth in a country that has negligible tourism sector in comparison with other economic sectors. The effect of uncertainty on growth is evident [5], and the temporal relationship between GDP and international tourism is relatively isolated [6]. Therefore, countries (or groups of countries) where tourism is a bigger proportion of GDP were not considered as a threshold variable in the present study, which examines a hybrid artificial intelligence (AI) model to forecast tourist arrivals to Taiwan from its top four markets.

Many researchers have proposed approaches to forecasting demand in the tourism industry. The most common time series methods include the autoregressive integrated moving average (ARIMA) model [7] and exponential smoothing (ETS) [8]. These methods usually employ historical datasets to forecast future tourist flow through a univariate or multivariate mathematical function that is highly dependent on linear assumptions. Although widely recognized, such methods are limited by their poor nonlinear fitting capabilities [9]. This indicates the usefulness of nonlinear methods of demand forecasting, namely, the use of artificial neural networks (ANNs) [10] and support vector regression (SVR) [11]. Nevertheless, the benefits of AI methods depend on using appropriate parameter settings. Various techniques have been proposed to determine an adequate set of parameter values; however, a lack of thorough guidelines remains a problem [12]. Moreover, researchers have widely applied several consecutive lagged variables as input features in forecasting problems. Nevertheless, some input features may be redundant or even irrelevant to a specific demand forecasting problem, which reduces the accuracy of forecasting models [13]. Therefore, in this study, the feature selection (FS) method is applied to identify essential data and improve the forecasting effectiveness of the input variables.

To develop a reliable forecasting tool for the tourism industry, this study proposes a hybrid algorithm called FS–PSOSVR, which is a combination of FS techniques, the SVR model, and the particle swarm optimization (PSO) algorithm. FS is used to determine the most relevant input variables in the time series data. The PSO algorithm is employed to determine a set of optimal parameters for SVR, which are then used to construct the SVR model. To account for regional differences, tourism demand forecasting models are constructed for different regions. Monthly data of tourist arrivals in Taiwan from January 2006 to December 2016 are used as an example. The experimental results demonstrated that the proposed algorithm outperformed other forecasting methods such as ETS, ARIMA, seasonal ARIMA (SARIMA), grid search SVR (GRIDSVR), and PSOSVR.

## 2. Literature Review

Coshall and Charlesworth suggested that the methods used by studies on forecasting tourism demand can be categorized into causal econometric models and time series models [14]. Causal econometric models attempt to establish relationships between variables such as tourism demand (as measured by the number of tourist arrivals at a destination) or tourist expenditure and a set of hypothesized explanatory factors. The most commonly used causal econometric models in the literature are cointegration and error correction models [15], vector autoregressive models [16], and linear almost-ideal system models [17]. These methods have also been combined [18].

In past decades, researchers have widely applied time series models, including ARIMA and ETS, to analyze issues concerning tourism demand forecasting. Lim and McAleer used ARIMA and SARIMA models to forecast the demand for tourism in Australia by analyzing data on tourists from Hong Kong, Malaysia, and Singapore [19]. Their results demonstrated that ARIMA is the most suitable model for predicting tourism demand for visiting Australia by visitors from Hong Kong and Malaysia. However, for Singaporean visitors, the SARIMA model generated superior results.

Chu used nine time series models—including two naive models [20], ARIMA-type models (ARIMA, SARIMA, and ARFIMA) and regression-based models—to forecast the volume of monthly tourist arrivals in Singapore. Chu reported that the ARFIMA model exhibited the highest forecasting accuracy both in the short and long term; however, for the medium term, the SARIMA model had superior performance [20].

More recently, Wan et al. used the SARIMA model and compared it with a seasonal moving average model and the Holt–Winter model [21]. Their findings indicated that the SARIMA model performed most favorably under all three h-step-ahead forecasting horizons. In addition, Baldigara and Mamula employed the SARIMA model to predict the number of German visitors to Croatia [22]. In their analysis, the predictive power of the SARIMA model was superior to that of the other methodologies mentioned in their study.

Assaf et al. used a comprehensive and accurate systematic approach to analyze tourism demand that was based on a Bayesian global vector autoregressive model [23]. Huang et al. used the PSO algorithm combined with a back-propagation neural network to establish a demand estimation model [24]. Akin et al. considered the SARIMA, *ν*-SVR, and multilayer perceptron-type neural networks and optimized network parameters using numerous approaches for evaluating performances on monthly tourist arrival data to Turkey from various countries [25]. Cang proposed a nonlinear combination method using multilayer perceptron neural networks to map the nonlinear relationship between inputs and outputs [26]. Huarng et al. proposed an innovative forecasting model to detect regime switching properly and used a fuzzy time series model for forecasting [27].

SVR overcomes classification problems, nonlinear function problems, and forecasting problems by using loss functions. Because tourism data usually display nonlinear characteristics, SVR is also widely applied in research on tourism demand forecasting. Chen and Wang combined SVR with a genetic algorithm (GA) to forecast the number of inbound visitors to China [28]. Their results illustrated that the forecast generated through GA–SVR was more persuasive than the forecasts of back-propagation neural network and ARIMA models. Hong et al. combined a chaotic GA with an SVR model to obtain forecasting information of visitor arrivals to Barbados [29]. This combined method achieved a more accurate estimation than the other models.

Most recently, Tsaur and Chan suggested that gray SVR can be employed to forecast the number of visitors from China to Taiwan [30]. The results obtained using this proposed method were superior to those obtained using other methods such as regression analysis, simple ETS, and a gray model.

## 3. Methodology

### 3.1. SVR

Support vector machines (SVMs) were initially introduced to address classification problems [31]. SVR is a version of an SVM and was proposed by Drucker et al. [32]. The basic functions of the SVR model are designed to provide a nonlinear mapping function that maps the training data to a high-dimensional feature space. The training dataset is denoted by {(*x*_*i*_, *y*_*i*_); *i*=1,2,…, *N*; *x*_*i*_ ∈ *R* ^*n*^; *y*_*i*_ ∈ *R*}, where *x*_*i*_ is the *i*th input in the *n*th dimension, *y*_*i*_ is the actual output, and *N* is the data set size. The SVR function is then

(1) $$\begin{array}{c} y=f\left(x_{i}\right)=w^{T}\varphi \left(x_{i}\right)+b, \end{array}$$

where *f*(*x*) denotes the forecast values, *φ*(*x*) is the feature function of the inputs, and *w* and *b* are adjustable coefficients. Through employing a penalty function to estimate the values of coefficients *w* and *b*, the penalty function *R*(*C*) becomes

(2) $$\begin{array}{c} R\left(C\right)=\frac{1}{2}{\left\|w\right\|}^{2}+C\cdot \frac{1}{n}\overset{n}{\underset{i=1}{\sum }}{\left|y_{i}-f\left(x\right)\right|}_{\varepsilon }, \end{array}$$

(3) $$\begin{array}{c} {\left|y-f\left(x\right)\right|}_{\varepsilon }=\left\{\begin{array}{cc} 0, & \left|y-f\left(x\right)\right|\leq \varepsilon ,\\ \left|y-f\left(x\right)\right|-\varepsilon , & \text{otherwise,} \end{array}\right. \end{array}$$

where *C* is the penalty coefficient and *ε* is the maximum value of tolerable error [33]. Two slack variables *ξ*_*i*_ and *ξ*_*i*_^*∗*^ are introduced to cope with the infeasible constraints of the optimization problem, which becomes

(4) $$\begin{array}{c} \underset{\omega ,b,\xi^{\left(*\right)}}{\min}\frac{1}{2}{\left\|w\right\|}^{2}+C\overset{n}{\underset{i=1}{\sum }}\left(\xi_{i}+\xi_{i}^{*}\right),\\ \text{subject to}\left\{\begin{array}{cc} -y_{i}+w^{T}\varphi \left(x_{i}\right)+b\leq \varepsilon +\xi_{i}, & \left(i=1,\ldots ,m\right),\\ y_{i}-w^{T}\varphi \left(x_{i}\right)-b\leq \varepsilon +\xi_{i}^{*}, & \left(i=1,\ldots ,m\right),\\ \xi_{i}^{\left(*\right)}\geq 0, & \left(i=1,\ldots ,m\right), \end{array}\right. \end{array}$$

where *ξ* ^(*∗*)^ ensures that the constraint is satisfied, *C* controls the balance between model complexity and training error rate, and *ε* is a constant for controlling the tube size; if *ε* is too small, overfitting may occur, and the opposite situation may cause underfitting. Using the Lagrange equation, the dual optimization problem is obtained:

(5) $$\begin{array}{c} \underset{\alpha_{i},\alpha_{i}^{*}}{\min}\frac{1}{2}\overset{n}{\underset{i,j=1}{\sum }}y_{i}\left(\alpha_{i}-\alpha_{i}^{*}\right)\left(\alpha_{j}-\alpha_{j}^{*}\right)k\left(x_{i},x_{j}\right)+\overset{n}{\underset{i=1}{\sum }}\left(\left(\varepsilon -y_{i}\right)\alpha_{i}+\left(\varepsilon +y_{i}\right)\alpha_{i}^{*}\right),\\ \text{subject to}\left\{\begin{array}{cc} \overset{N}{\underset{i=1}{\sum }}\left(\alpha_{i}-\alpha_{i}^{*}\right)=0, & \\ 0\leq \alpha_{i}^{\left(*\right)}\leq C, & \left(i=1,\ldots ,m\right). \end{array}\right. \end{array}$$

To solve (5), the SVR function can be obtained as follows:

(6) $$\begin{array}{c} f\left(x\right)=\overset{n}{\underset{i=1}{\sum }}\left(\alpha_{i}-\alpha_{i}^{*}\right)k\left(x_{i},x\right)+b, \end{array}$$

where *α*_*i*_ and *α*_*i*_^*∗*^ are the Lagrange multipliers and *k*(*x*_*i*_, *x*) is a kernel function. The kernel function constructs a nonlinear decision hypersurface in the SVR input space. The most widely used kernel, the Gaussian radial basis function (RBF) kernel, not only performs nonlinear mapping between the input space and a high-dimensional space but is also easy to implement, and thus, it is suitable for solving nonlinear problems. Therefore, the Gaussian RBF kernel was employed in this study:

(7) $$\begin{array}{c} k\left(x_{i},x\right)=\exp\left(-\sigma {\left\|x-x_{i}\right\|}^{2}\right), \end{array}$$

where *σ* represents the scaling factor of the Gaussian RBF kernel.

### 3.2. Particle Swarm Optimization

PSO is a population-based iterative optimization algorithm inspired by the social behavior of bird flocking that was developed by Eberhart and Kennedy [34]. PSO has successfully been applied in numerous researches [35–37]. The optimization process starts with a randomly initialized population of solutions, which are called particles. The swarm consists of *n* particles, and each particle has a position vector *x*_*i*_=(*x*_*i*,1_,…, *x*_*i*,*d*_) and velocity vector *v*_*i*_=(*v*_*i*,1_,…, *v*_*i*,*d*_), where *i*=1,2,…, *n* and *d* are the number of dimensions in the vector. Each particle is a potential solution to the problem in the *D*-dimensional search space. The particles share information with each other; thus, each particle can be influenced to adjust their search direction toward a promising search region. Each particle has its own optimal experience, represented as the best known position of particle *i* (*pbest*_*i*_) in the feature space, and the optimal experience derived from the population is represented as the best known position within the population (*gbest*). During each generation, each particle is accelerated toward *pbest*_*i*_ and *gbest*. The value of experience is evaluated using the fitness function *f*(*x*) according to the problem definition. Both position and velocity must be limited to between the rational lower boundary *b*_low_ and upper boundary *b*_up_ in the feature space. The updated velocity and position can be obtained using the following equations:

(8) $$\begin{array}{c} v_{i,d}^{\text{new}}=wv_{i,d}^{\text{old}}+c_{1}r_{1}\left(pbest_{i,d}-x_{i,d}\right)+c_{2}r_{2}\left(gbest_{d}-x_{i,d}\right), \end{array}$$

(9) $$\begin{array}{c} x_{i}^{\text{new}}=x_{i}^{\text{old}}+v_{i}^{\text{new}}, \end{array}$$

where *w* is the inertia weight, *c*_1_ and *c*_2_ are acceleration constants, and *r*_1_ and *r*_2_ are uniformly random values between 0 and 1.

The inertia weight *w* controls the current velocity. A larger inertia weight facilitates global exploration, whereas a small value promotes local exploration. To balance global and local exploration capabilities; this study employs the commonly used linearly decreasing inertia weight (LDW), because an inertia weight that decreases with time from 0.9 to 0.4 is more favorable than a fixed inertia weight. The LDW formulation is as follows:

(10) $$\begin{array}{c} w=w_{\min}-\left(w_{\max}-w_{\min}\right)\times \frac{\text{iter}}{\max\_\text{iter}}, \end{array}$$

where *w*_max_ is set at 0.9, *w*_min_ is set at 0.4, and max_iter and iter are the maximum iteration and current iteration, respectively.

All operations in PSO are repeated until the termination condition is reached. The termination condition corresponds to the maximum number of iterations. The PSO algorithm is described in Algorithm 1.

### 3.3. Selecting the SVR Parameters Using PSO

**Figure 1:**
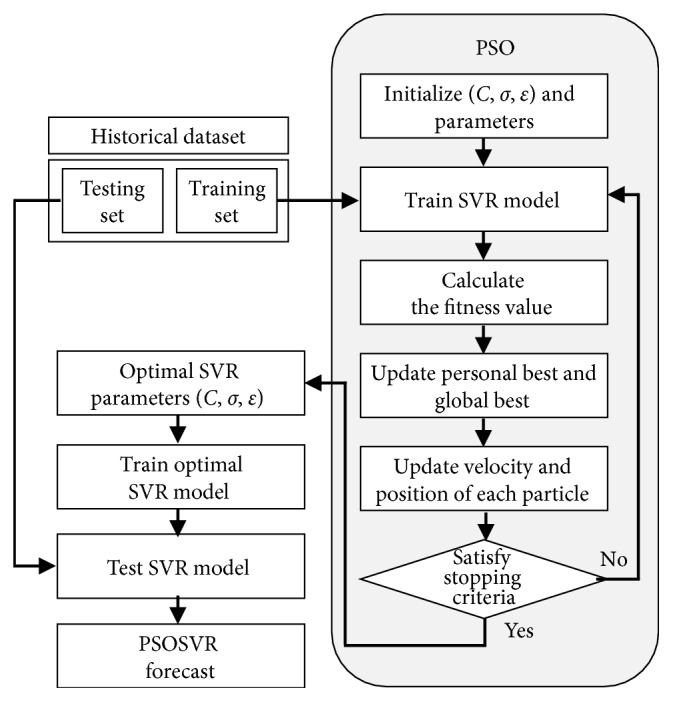
Flowchart of PSOSVR.

In SVR modeling, the parameter settings affect the performance of a forecast time series, as mentioned in the preceding discussion of PSO. The crucial parameters are the regularization parameter (*C*), bandwidth of the kernel function (*σ*), and tube size of the *ε*-insensitive loss function (*ε*). Improper choice of parameter values can result in either overfitting or underfitting [38]. Consequently, selecting the optimal parameters is crucial when employing SVR to forecast a time series. In this study, the PSO algorithm is utilized to select approximations of the three parameters of the SVR model.  Figure 1 presents a flowchart of PSOSVR.

The procedures of the PSOSVR model are as follows:

*Step 1: initialization*. First, the initialization values of the parameters are set; then, the particles are generated in the feature space. Each particle *i* is represented by *x*_*i*_ = {*C*, *σ*, *ε*}.

**Figure 2:**
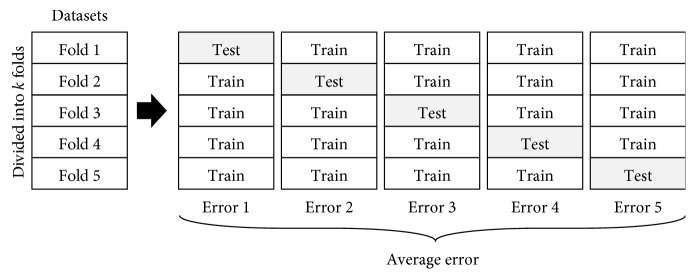
Concept of *k*-fold CV (*k* = 5).

**Figure 3:**
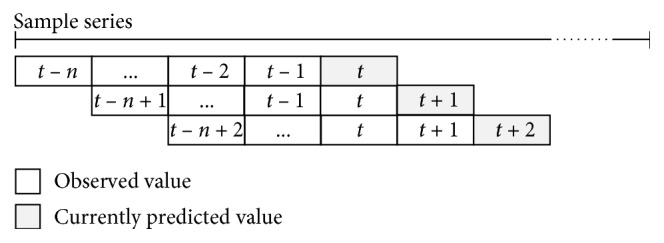
Rolling-based forecasting mechanism.

*Step 2: fitness evaluation*. After the encoding procedure is completed, the three values of parameters *C*, *σ*, and *ε* are inserted into the SVR model to forecast the problem; a *k*-fold cross-validation (CV) is employed in the training phase to avoid overfitting, and the validation error is calculated.  Figure 2 illustrates the concept of *k*-fold CV. The PSOSVR model uses a rolling-based procedure to forecast data.  Figure 3 illustrates the rolling-based mechanism. The previous 12 lagged observation data points are selected as input variables and the current data as output variables. First, the top 12 tourist datasets are fed into the proposed model. Following this, a one-step-ahead forecasting value is obtained. The next rolling 12 data points are fed into the proposed model again, and the second forecasting value is obtained. The process is repeated until all the forecasts in the training set are obtained, following which the validation error is calculated. In this study, the mean absolute percentage error (MAPE) is adopted as the fitness function. The MAPE is calculated using the following:

(11) $$\begin{array}{c} {\text{MAPE}}_{\text{CV}}=\frac{1}{N}\overset{N}{\underset{i=1}{\sum }}\left|\frac{y_{i}-f_{i}}{y_{i}}\right|\times 100\%, \end{array}$$

where *y*_*i*_ is the actual value, *f*_*i*_ is the forecast value, and *N* is the sample size.

*Step 3: update of pbest*. If the fitness value of particle *i* in the current iteration exceeds that of *pbest*_*i*_, then *pbest*_*i*_ is replaced by *x*_*i*_.

*Step 4: update of gbest*. If the fitness value of *pbest*_*i*_ in the current iteration exceeds that of *gbest*, then *gbest* is replaced by *pbest*_*i*_.

*Step 5: update of velocity*. The velocity of each particle is calculated according to (8).

*Step 6: update of position*. The position of each particle is calculated according to (9).

*Step 7: stop criteria*. The processes are repeated in the aforementioned order until the maximum iteration is reached.

### 3.4. Random Forest

Random forest (RF) is an ensemble learning method for both classification and regression problems [39]. The principle of RF is to combine a set of binary decision trees, each of which is constructed using a bootstrap sample obtained from the learning sample and a subset of features (input variables or predictors) randomly chosen at each node. The prediction is made using a majority vote of the trees (in classification) or by averaging their outputs (in regression). In addition to classification and regression, RF provides an internal measure of variable importance through computing importance scores. Similarly, it can be used to select crucial features. During the construction of an RF, each node of a decision tree is split into two children, whereas a splitting criterion is used to reduce the impurity of a node, which is measured through Gini importance [39]. In the process of node splitting, *i* is the impurity of the node, and the node's Gini importance is defined as follows:

(12) $$\begin{array}{c} i=1-\underset{j}{\sum }p^{2}\left(j\right), \end{array}$$

where *p*(*j*) is the proportion of samples that are labeled *j* in this node. After splitting, the impurity of the node is described as follows:

(13) $$\begin{array}{c} \Delta i=i_{\text{parent}}-\left(p_{\text{left}}\times i_{\text{left}}+p_{\text{right}}\times i_{\text{right}}\right), \end{array}$$

where *p*_left_ and *p*_right_ are the sample proportions of the left and right child nodes, respectively, and *i*_parent_, *i*_left_, and *i*_right_ are the Gini importances of the parent, left child node, and right child node, respectively. For any one feature *X*_*i*_, the sum of its impurity decrement in all decision trees is the Gini importance of *X*_*i*_:

(14) $$\begin{array}{c} \alpha \Delta I=\underset{k}{\sum }\Delta i_{k}. \end{array}$$

This equation indicates the importance of each feature, and a greater value indicates that the feature is more important.

Recursive feature elimination (RFE) is a recursive process based on feature ranking techniques [40]. According to a certain feature ranking standard, RFE starts from a complete set and then eliminates the least relevant feature one at a time to select the most important features. This study uses an FS method that combines RFE and RF, named, RF–RFE. The process is presented in Algorithm 2.

### 3.5. FS–PSOSVR

**Figure 4:**
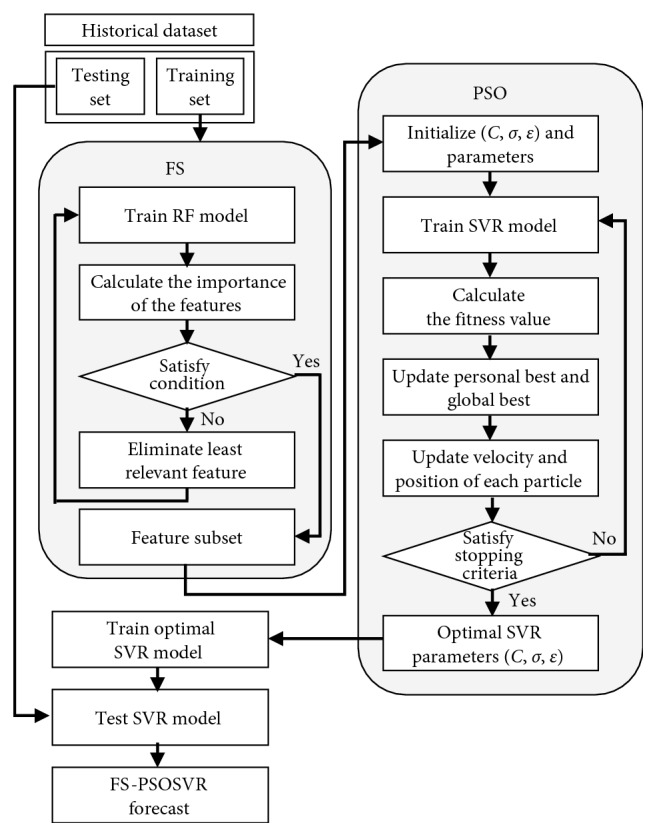
Flowchart of FS–PSOSVR.

The FS–PSOSVR model is proposed to determine the most effective feature subset and improve the forecasting performance of PSOSVR.  Figure 4 illustrates the algorithm, and the detailed steps are as follows:

*Step 1*. the data set is divided into a training set and a test set. The training set is used as the original subset *F.*

*Step 2*. the RF model is trained using subset *F*, and the variable importance scores of each feature in the subset are calculated.

*Step 3*. the least important feature is eliminated from *F*, and Step 2 is repeated until the desired number of features is obtained.

*Step 4*. after allowing the new training set to be the feature subset *F* obtained by RF–RFE, the PSOSVR process is initiated.

### 3.6. Performance Criteria

Two common statistical metrics, root-mean-square error (RMSE) and MAPE, are used to evaluate the performance of the forecasting models (Table 1) by comparing the deviation between the real and forecast values. Lower RMSE and MAPE values represent higher accuracy, thus indicating that the forecast values are reliable. Lewis [41] developed a table (Table 2) containing typical MAPE values for analyzing and interpreting industrial and business data.

### 3.7. Parameter Settings

The size of the population is set at 50; acceleration factors *c*_1_ and *c*_2_ are both set at 2.0; and the maximum number of iterations (max_iter) is set at 100. These parameters are selected according to Bratton and Kennedy [42]. In this study, the search scopes of the SVR parameters are set at *C* = [2^0^, 2^10^], *σ* = [2^−8^, 2^0^], and *ε* = [2^−8^, 2^0^]. The traditional SVR model uses the grid search method (GRIDSVR) to determine the optimal parameters. GRIDSVR increments the parameters exponentially; thus, the search spaces of GRIDSVR are set as *C* = [2^0^, 2^1^, 2^2^,…, 2^10^], *σ* = [2^−8^, 2^−7^, 2^−6^,…, 2^0^], and *ε* = [2^−8^, 2^−7^, 2^−6^,…, 2^0^].

## 4. Results and Discussion

The auto.arima and ets functions of the *R* forecast package [43] were used to identify (S)ARIMA and ETS models. The *Python* module sklearn.svm, which is an interface of the LIBSVM library [44], was used to train the SVR-based models.

### 4.1. Data Sets and Preprocessing

**Figure 5:**
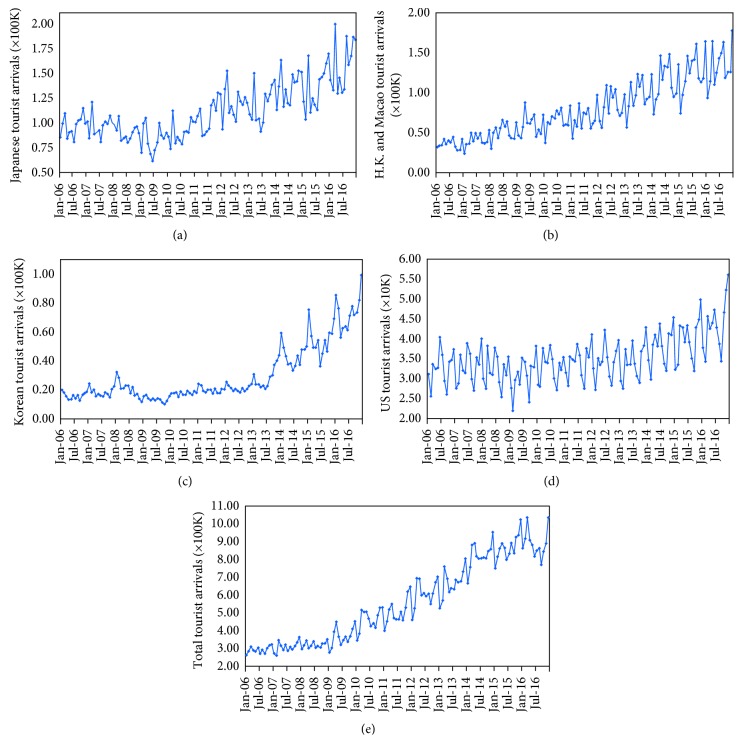
Monthly tourist arrivals from January 2006 to December 2016 from (a) Japan, (b) Hong Kong and Macao, (c) South Korea, (d) the United States, and (e) the total number.

This paper presents a hybrid AI model to forecast tourist arrivals to Taiwan from the top four markets. To evaluate the proposed approach, we applied it to data on tourist arrivals to Taiwan that have been used in several papers [6, 45–47] as a case study. Specifically, data for monthly tourist arrivals to Taiwan from 2006 to 2016, collected from the Tourism Statistics Database [48], were used. Japan, Hong Kong and Macao, South Korea, and the United States (Figure 5) were selected as the four groups that contributed the most visitors to Taiwan. Visitors from China were excluded because drastic fluctuations often occur as a result of the cross-strait relationship and political uncertainty. Each data set was divided into two subsets:

*Training set*: used for training the model; it consisted of the monthly data for 2006–2015.

*Test set*: used for testing the forecast accuracy; it consisted of the monthly data for 2016.

Hsu et al. [49] noted that scaling original data helps improve forecast performance and predictive accuracy by not allowing attributes with greater numerical ranges to dominate those with smaller numerical ranges and avoiding potential numerical problems. In this study, each data set was normalized within the range [0.15, 0.85] using the following:

(15) $$\begin{array}{c} \tilde{X_{t}}=\frac{X_{t}-X_{\min}}{X_{\max}-X_{\min}}\times 0.7+0.15, \end{array}$$

where *X*_*t*_ is the number of tourist arrivals at time *t* and *X*_max_ and *X*_min_ are the maximum and minimum numbers of tourist arrivals in the period of the data set, respectively.

### 4.2. Comparison of Time Series Models and SVR-Based Models

**Figure 6:**
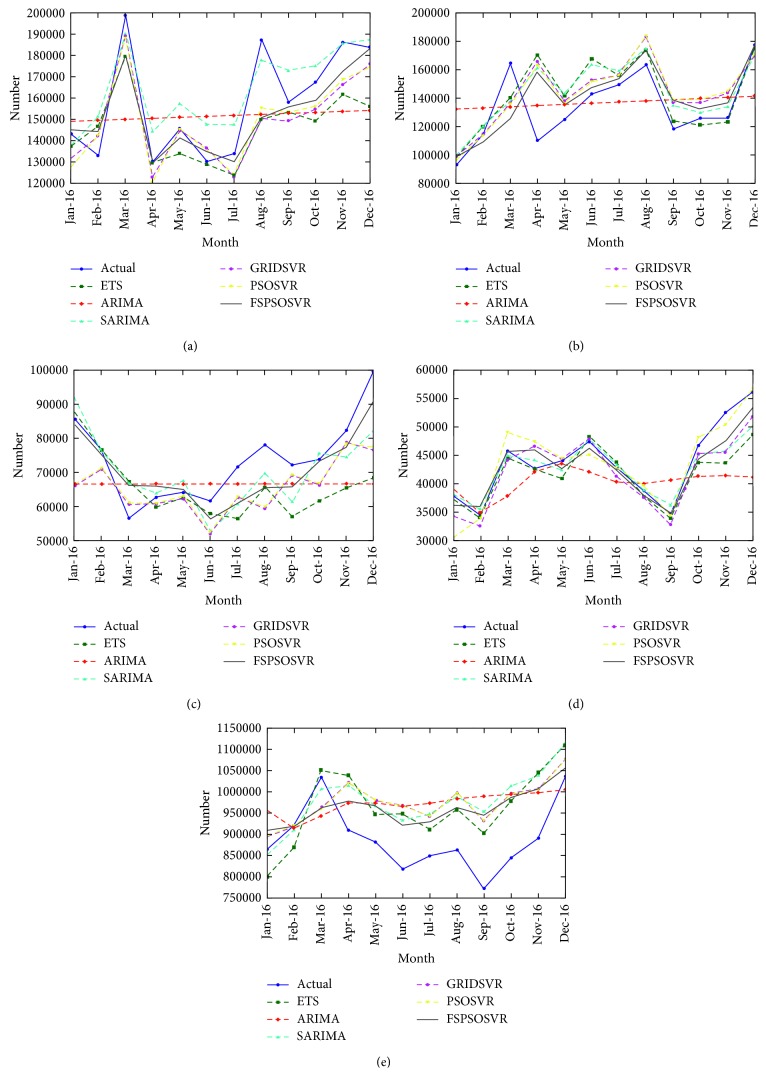
Forecast results for different datasets: (a) Japan; (b) Hong Kong and Macao; (c) South Korea; (d) the United States; and (e) total tourist numbers.

Figure 6 depicts the differences between the actual data and forecast results. These figures reveal that the results obtained using the proposed FS–PSOSVR method more accurately reflect the actual data compared with the results obtained using the other methods. The MAPE and RMSE for each method in Table 3 were also employed to compare forecast performances. To test and verify the forecasting ability of the AI model for the time series model, the ETS, ARIMA, and SARIMA models were set as the objects of comparison. Compared with the time series model, instead of confirming whether the data belong to a stationary state and considering whether other statistical tests should be applied, the AI model learns from characteristics of the training data.  Table 4 lists the average MAPE values of the forecasts obtained using ETS, ARIMA, SARIMA, GRIDSVR, PSOSVR, and FS–PSOSVR. For the time series forecasting problem, the AI model demonstrated a similar ability to the time series model. Moreover, the FS–PSOSVR method was superior in solving the forecasting problems.

### 4.3. Comparison of GRIDSVR, PSOSVR, and FS–PSOSVR

This study measured the ability to obtain the optimal parameters of GRIDSVR and PSOSVR to prove that the effectiveness of the SVR method depends on the parameters selected. The method that GRIDSVR employs to reach the most suitable parametric combination is a grid search, which involves calculating the fitness value of each grid. This may indicate that the most suitable parameter combinations do not exist in the grid; therefore, PSO was used to improve analysis of the problem.  Table 5 presents the optimal values of the three SVR parameters for each SVR-based model. The results obtained using PSOSVR were more accurate than those obtained using GRIDSVR (Figure 6). This observation indicates that PSO achieved more favorable forecasting results.

To increase forecasting accuracy, the FS method was employed. RF–RFE was used to identify the reliable lagged variables. To determine the appropriate number of features, this study tested four to eight features to determine which number was optimal. The lagged variables are presented in Table 6, and *y*_*t−i*_ indicates the number of visitors *i* months ago. The results indicated that after applying FS, the prediction ability was superior to that of PSOSVR without FS. Additionally, by removing the input variable with less influencing power, a more suitable result was obtained.

### 4.4. Analysis for Individual Data

#### 4.4.1. Japan

Increasing the accuracy of forecasting is especially helpful for Japanese tourists because the tourism market from Japan has remained steady. The MAPE value of each forecasting model was less than 10; according to the interpretation of MAPE values by Lewis [41], all MAPE results can be categorized as denoting high accuracy. For the FS–PSOSVR method, the greatest gap between the experimental results and real data occurred in the data from August 2016. Based on the data from Taiwan's Tourism Bureau, compared with the growth rate in 2015—which remained constant—the growth rate for the entire year of 2016 increased significantly to 16.5%. Among the data for each month, the growth rate of Japanese visitors traveling to Taiwan increased to approximately 30%. This dramatic growth is the main reason that the experimental outcomes do not match the forecast [50]. Although the SARIMA model obtained results that were more similar to the real numbers in March and August 2016, causing their RMSE values to decrease, the holistic results were still slightly higher than the actual results. This suggests that the SARIMA model does not accurately reflect the actual tourist trend.

#### 4.4.2. Hong Kong and Macao

As with the results for Japan, all MAPE results for Hong Kong and Macao indicated high accuracy. The number of visitors from Hong Kong and Macao in March and April is influenced by the Easter vacation. Every model used by this study suggested that the number of tourists increases in April but decreases in May. In 2016, the Easter vacation, occurring in March, was the external factor. This external factor—the variable dates of the Easter vacation—is not accounted for in the univariate analysis. In contrast to August and September of the previous year (2015), the growth rate in 2016 slightly increased (1.75% for August and 0.35% for September). This reveals that most of the models used in this study overestimated visitors from Hong Kong and Macao, and they are therefore unsuitable.

#### 4.4.3. South Korea

Only the SARIMA and FS–PSOSVR models reached the standard of high accuracy for South Korea. The other models achieved adequate results, but substantial differences between each model's results were observed. Differences in tourism levels can be inferred from changing government policies. In the past 3 years, the Taiwanese government has commissioned more advertisements to promote tourism. Compared with the statistics from 3 years prior to that, the number of South Korean tourists rose considerably. This noticeable growth produces deviations that influenced the final forecasting results. Because of the SVR model's ability to learn from historical data, the differences that GRIDSVR and PSOSVR obtained were markedly larger than those obtained using other models from August, September, and December of 2016. Nonetheless, in contrast to the forecasting results obtained using the other two SVR models, FS–PSOSVR—which selects the features with higher influence authority in advance—obtained an accurate result.

#### 4.4.4. United States

All the MAPE results indicated high accuracy; this is because the tourism market of the United States has remained steady over the last decade. Visible fluctuations occurred in April, November, and December of 2016. In the past two years, the number of American visitors increased dramatically in April—by 22.03% in 2015 and 4.95% in 2014—even though the total number of visitors in 2016 decreased by 0.84%. For November and December, the real data indicated rises of 16.71% and 12.66%, respectively, which were much higher than those observed in 2015 (8.99% and 9.87%) and 2014 (7.36% and 5.55%). Consequently, a larger discrepancy occurred between the real and predicted numbers.

#### 4.4.5. Total

From July 2016, the total number of visitors increased slowly, mainly because the number of visitors from China decreased dramatically. Notably, the statistics for September suggested much fewer visits than the previous year. This suggests that policy changes and political events have a dramatic effect on the willingness and ability of tourists from China to travel to Taiwan. The influence of policies is an external factor that cannot be predicted in the univariate analysis. For all tourists, as for those from Hong Kong, the Easter vacation had a strong influence, with the most obvious discrepancy occurring in March and April. This demonstrates that the model cannot identify a steady mode during the training process, leading to apparent differences.

Because tourists from different countries may perceive a destination differently because of unique motivations and expectations [51], effective policy interventions can be implemented to solve this problem. Furthermore, policy interventions can guidance for tourism planning and development, thereby creating a favorable tourism environment [52]. Also crucial for effective tourism planning is maintaining the quality of tourists' experiences and shaping their perceptions of places and lifestyles [52, 53]. To increase the number of visitors to Taiwan, the government and major tour operators must tailor their marketing efforts to each individual territory and strive to construct and maintain Taiwan's positive image using various methods.

Lin and Kuo [54] asserted that the concept of nationality implies notions of cultural values, social norms, and economic development. Given the dynamic effect of nationality, travel agencies could improve their inclusive group packages to generate diversified images. Moreover, a culture of cooperation between different and heterogeneous actors in the business value chain should be fostered [52]. Another necessary approach for Taiwan's destination managers to expand their market reach is to explore potential products that may lure newly emerging markets to Taiwan, such as the younger generation Y segment, while continuing to attract the older Baby Boomer generation [55].

Taiwan's policy makers and destination managers must also acknowledge market diversity to devise more specific strategies for different market segments and design desirable destination experiences to attract more tourists throughout the year [56]. Moreover, to provide a deeper level of engagement with destinations, service providers must offer a wide range of services and resources to tourists in order to enable them to fully enjoy their stay, thereby positively affecting their evaluation of Taiwan as a destination [53]. Destination managers in Taiwan must develop their capacity and capability to deliver new and innovative products and services to leverage the full potential of Taiwan as a tourist destination [57].

### 4.5. Advantages and Limitations

The results show that FS–PSOSVR performs superior forecasting of tourist arrivals. FS–PSOSVR retains the advantages of FS, PSO, and SVR. There are no additional parameters in the PSO and no limits to the number of constraints. During the numerous iterations, the most optimal particle transmits the information onto other particles; thus, FS–PSOSVR has fast re-searching speed [58]. Furthermore, PSO has no overlapping and mutation calculations; thus, the search can be conducted at the particle speed. FS–PSOSVR also has advantages in high dimensionality space because SVR does not depend on the dimensionality of the input space. The advantage of feature space representation in tourist arrival forecasting is the mean squared error as well as that the loss function is also the mean squared error [32]. The FS in FS–PSOSVR can reduce noise to improve forecasting accuracy; furthermore, more interpretable features can facilitate understanding of the importance of features. However, FS–PSOSVR has some limitations in terms of convergence speed because PSO easily suffers from partial optimism, which causes less accuracy in terms of regulation of its speed and direction. The SVR model is a well-known forecasting approach that has been applied to solve time series problems [59]. Our results also demonstrated that FS–PSOSVR was superior to SARIMA in terms of forecasting tourist arrivals. However, FS–PSOSVR does not address how to handle seasonal time series datasets. Thus, we suggest applying decomposition techniques to obtain decomposed seasonal time series data when a data set belongs to seasonal time series data [60].

## 5. Conclusion

Forecasting of tourist arrivals is critical to accurately predict requirements for infrastructure development. In this study, we proposed an FS–PSOSVR algorithm for the forecast of tourism demand. In FS–PSOSVR, FS is used to identify essential data and improve the SVR forecasting effectiveness of input variables. We applied PSO to tune the suitable parameters for SVR and more effectively forecast tourism demand. The predictive power of the method was compared with that of five forecasting models: ETS, ARIMA, SARIMA, GRIDSVR, and PSOSVR. The parameters acquired by FS–PSOSVR were more accurate than the parameters derived from GRIDSVR and PSOSVR, indicating that FS–PSOSVR is more effective at optimizing the parameters of SVR than GRIDSVR and PSOSVR are. Moreover, FS–PSOSVR achieved greater forecasting accuracy than other methods such as ARIMA, SARIMA, and ETS, indicating that FS–PSOSVR is a relatively more effective means of forecasting tourism demand.

## Figures and Tables

**Algorithm 1:**
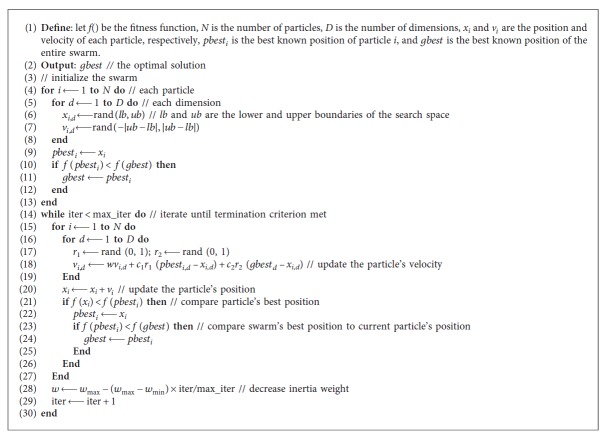
Particle swarm optimization algorithm.

**Algorithm 2:**
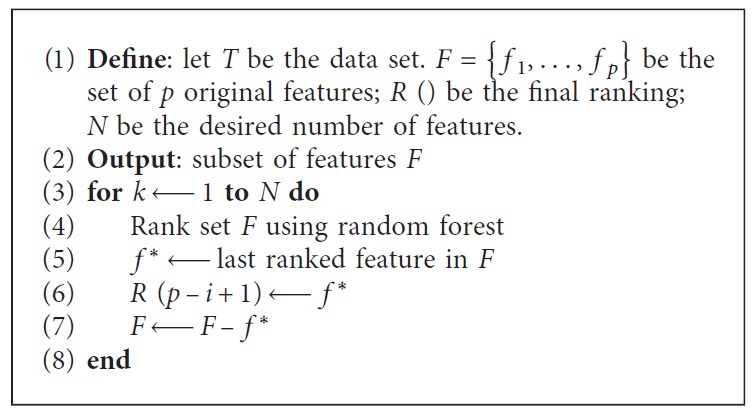
Random forest—Recursive feature elimination (RF–RFE).

**Table 1:** Performance metrics.

Metrics	Calculation
RMSE	$\sqrt{\left(1/N\right)\sum_{i=1}^{N}{\left(y_{i}-f_{i}\right)}^{2}}$
MAPE	(1/*N*)∑_*i*=1_^*N*^|(*y*_*i*_ − *f*_*i*_)/*y*_*i*_| × 100%

*y*_*i*_ is the actual value, *f*_*i*_ is the forecast values, and *N* is the sample size.

**Table 2:** Interpretation of MAPE values.

MAPE	Interpretation
<10%	Highly accurate forecasting
10–20%	Good forecasting
20–50%	Reasonable forecasting
>50%	Inaccurate forecasting

**Table 3:** Forecast tourist arrivals obtained using ETS, ARIMA, SARIMA, GRIDSVR, PSOSVR, and FS–PSOSVR.

Case		ETS	ARIMA	SARIMA	GRIDSVR	PSOSVR	FS–PSOSVR
Japan	MAPE (%)	8.54	12.87	7.25	7.22	6.95	**5.23**
	RMSE	18091.23	24665.02	**11878.9**	14670.95	13616.38	13308.91

Hong Kong and Macao	MAPE (%)	11.07	16.28	12.10	12.39	12.24	**10.65**
	RMSE	21045.26	23224.72	20043.2	21168.17	21013.52	**19738.13**

South Korea	MAPE (%)	13.48	13.58	9.91	11.45	11.14	**7.66**
	RMSE	13482.26	13515.24	8681.24	11439.02	11137.59	**6807.39**

The United States	MAPE (%)	4.79	10.03	3.95	5.45	4.62	**3.84**
	RMSE	3626.19	6508.72	2786.49	3027.27	2883.04	**2218.12**

Total	MAPE (%)	10.64	12.08	11.24	11.21	11.14	**9.76**
	RMSE	100954.40	116687.03	111400.6	108457.15	107765.04	**95910.99**

Bold: the superior values.

**Table 4:** Average MAPE of ETS, SARIMA, GRIDSVR, PSOSVR, and FS–PSOSVR.

	ETS	ARIMA	SARIMA	GRIDSVR	PSOSVR	FS–PSOSVR
MAPE (%)	9.70	12.97	8.89	9.54	9.22	**7.43**

Bold: the lowest average MAPE.

**Table 5:** Training results of GRIDSVR, PSOSVR, and FS–PSOSVR.

Model	Data set	*C*	*ε*	*σ*
GRIDSVR	Japan	1024.00	0.0313	0.0156
	Hong Kong and Macao	128.00	0.0039	0.0313
	South Korea	1024.00	0.0156	0.0039
	The United States	128.00	0.2500	0.0625
	Total	256.00	0.0039	0.0039

PSOSVR	Japan	805.03	0.0423	0.0130
	Hong Kong and Macao	209.96	0.0039	0.0367
	South Korea	776.87	0.0159	0.0039
	The United States	415.75	0.0467	0.0367
	Total	216.66	0.0039	0.0039

FS–PSOSVR	Japan	564.45	0.1371	0.0241
	Hong Kong and Macao	389.08	0.0039	0.0039
	South Korea	673.88	0.0222	0.0094
	The United States	531.61	0.0268	0.0405
	Total	1016.71	0.0087	0.0060

**Table 6:** Lagged variables of FS–PSOSVR.

Data set	Lagged variables
Japan	*y*_*t*−12,_ *y*_*t*−3,_ *y*_*t*−2,_ *y*_*t*−1_
Hong Kong and Macao	*y*_*t*−12,_ *y*_*t*−4,_ *y*_*t*−3,_ *y*_*t*−1_
South Korea	*y*_*t*−12,_ *y*_*t*−11,_ *y*_*t*−10,_ *y*_*t*−3,_ *y*_*t*−2,_ *y*_*t*−1_
The United States	*y*_*t*−12,_ *y*_*t*−6,_ *y*_*t*−3,_ *y*_*t*−1_
Total	*y*_*t*−12,_ *y*_*t*−8,_ *y*_*t*−4,_ *y*_*t*−3,_ *y*_*t*−2,_ *y*_*t*−1_

## Data Availability

The data for monthly tourist arrivals to Taiwan from 2006 to 2016 used to support the findings of this study have been deposited in the Tourism Statistics Database.
